# Editorial: Computational drug discovery of medicinal compounds for cancer management

**DOI:** 10.3389/fchem.2023.1343183

**Published:** 2023-12-07

**Authors:** Sibhghatulla Shaikh, Khurshid Ahmad, Mohammad Ehtisham Khan, Faez Iqbal Khan

**Affiliations:** ^1^ Department of Medical Biotechnology, Yeungnam University, Gyeongsan, Republic of Korea; ^2^ Department of Chemical Engineering Technology, College of Applied Industrial Technology, Jazan University, Jazan, Saudi Arabia; ^3^ Department of Biological Sciences, School of Science, Xi’an Jiaotong-Liverpool University, Suzhou, Jiangsu, China

**Keywords:** computational methods, drug discovery, cancer, medicinal compounds, druglikeness

Cancer remains a major public health concern, with it ranking as the leading cause of death worldwide. Despite significant advances in biotechnology, developing practical and innovative small-molecule drugs remains a hard, time-consuming, and costly process. This endeavor necessitates the collaboration of experts from a variety of disciplines, including computational biology, drug metabolism, and clinical research. Hence, there is a pressing need for novel drug development processes that save time and cost while improving overall efficiency. Computer-aided drug design (CADD) methodologies are becoming increasingly crucial in drug discovery, particularly in their ability to identify promising drug candidates cost-effectively. In this area of study, we launched a Research Topic in Frontiers in Chemistry journal titled “Computational Drug Discovery of Medicinal Compounds for Cancer Management.” This Research Topic attracted the interest of researchers, and a large number of manuscripts were submitted. Among these submissions, 15 Original Research articles have been published covering a wide range of CADD topics and elucidating advanced *in silico* methodologies applicable to the field of drug discovery.

The following is a summary of the published articles:

Dain Md Opo et al. used structure-based pharmacophore modeling and virtual screening to identify potentially natural lead compounds that can inhibit BRAF and thus inhibiting cancer. Promising candidate compounds targeting the upregulated BRAF gene have been identified using *in silico* drug design methodologies and computational tools. The study identified four potential compounds by utilizing these computational methods. The investigation suggests that these compounds may be useful against a range of cancers by specifically targeting the overexpressed BRAF gene.

Ashraf et al. presented a comprehensive study on potential inhibitors of Tyrosine Threonine Kinase (TTK), a target in a variety of human cancers including breast, colorectal, and thyroid carcinomas. They used computational techniques like 3D-QSAR modeling and structure-based alignment. The study identified key factors influencing compound activity (electrostatic, steric, HBA, HBD, and hydrophobic fields). New compounds with predicted binding modes and structural stability were designed using molecular docking and molecular dynamics (MD) simulations, displaying promising TTK protein binding affinity as potential TTK inhibitor candidates.

Li et al. investigated LSD1, a protein methylation oxidase linked to gene expression and tumor initiation. Compound 17i showed promise as an LSD1 inhibitor and anti-tumor agent, but solubility issues hampered clinical validation. A carrier-free 17i nano assembly made with DSPE-PEG2000 improved therapeutic efficacy. In CT-26 mice with colorectal tumors, these nano assemblies matched 17i′s cytotoxicity *in vitro* but had better therapeutic efficacy, anti-tumor immune response, and lower systemic toxicity. A novel drug nanoassembly method may improve the effects of poorly soluble anti-tumor compounds.

He et al. computationally screened a library of 700 antiviral compounds against the 3CL protease (3CLP) of human noroviruses. The researchers discovered three compounds that had higher binding energy with 3CLP than the control (Dipeptidyl inhibitor 7). These hits’ estimated physicochemical and ADME properties were in the favorable range. According to the findings, these compounds could be used as 3CLP inhibitors in the treatment of gastroenteritis.

Mert-Ozupek et al. conducted an insilico screening of bioactive compounds from Caulerpa spp. against the colorectal cancer enzymes glucose-6-phosphate dehydrogenase and 6-phosphoglutarate dehydrogenase. Caulerpin, monomethyl caulerpinate, and caulersin bind to these enzymes strongly. They proposed that the identified compounds be tested for their potential efficacy against enzymes in the pentose phosphate pathway.

Ahmad et al. investigated the anticancer and apoptotic effects of carvacrol (CAR) on C33A cervical cancer cells. The antiproliferative and apoptotic effects of CAR were mostly observed in C33A cervical cancer cells *in vitro*. ROS production in C33A mitochondria triggered a chain of events that resulted in mitochondrial apoptosis. CAR also influences extrinsic or death receptor pathway signaling. CAR inhibited hedgehog signaling, causing apoptosis and inhibiting cell proliferation in cervical carcinoma cells. These findings imply that CAR could be used to treat cervical cancer.

Guo et al. explored 422 targets and 29 active ingredients from Astragali Radix (AR) and Spreading Hedyotis Herb. They demonstrated how AR-SH reduces lung adenocarcinoma (LUAD) symptoms by targeting EGFR, MAPK1, and KRAS. Molecular docking and dynamics simulations showed that AR-SH’s main active components bind to the right proteins, especially EGFR, suggesting it is more effective than Gefitinib. These findings show that AR-SH can improve LUAD treatment and prognosis.

Almukadi et al. identified novel and efficacious therapeutics for PIM-1 kinase by employing structure-based and machine-learning approaches. Four potential molecules were discovered to modulate PIM-1. Additionally, the MD simulation study revealed that these compounds interacted with the PIM kinase stably.

Alsukaibi et al. investigated the phytochemical and biological properties of two date fruit cultivars from Saudi Arabia’s Ha’il region, Shishi M1 and Majdool M2. *In vitro*, both cultivars inhibited HCT-116 colon cancer cells. Procyanidin B2 and luteolin-7-O-rutinoside were identified as active constituents by computational analysis.

Ali et al. used insilico techniques to predict how rare genetic variations would affect HRAS protein function. Fifty nonsynonymous single nucleotide polymorphisms (nsSNPs) were discovered, 23 of which were in the HRAS gene exon, implying that they are potentially harmful. Ten of the twenty-three tested substances were the most dangerous. This study lends credence to the notion that nsSNPs may increase HRAS expression and activate carcinogenic signaling pathways in cancer.

Ali et al. utilized phytocompounds targeting TP53 from *Amomum subulatum* seed extract, focusing on major alkaloids and saponins. The antioxidant activity was confirmed by DPPH analysis, particularly in methanol, BHT, and n-hexane extracts. Additionally, the computational analysis revealed that top phytocompounds had strong binding affinities to TP53, implying potential anti-cancer actions. This study presents novel cancer treatment drug discovery insights using *A. subulatum* seed compounds.

Binsaleh et al. investigated the effect of depression on breast cancer and prostate cancer patients during the COVID-19 pandemic. Cancer patients, especially those suffering from depression, had higher levels of proinflammatory cytokines and oxidative stress markers than healthy people. Elevated levels of specific serum antibodies in cancer patients suggested increased oxidative stress. The findings highlight the importance of addressing mental health concerns in cancer patient care and disease management.

Rashid et al. investigated the potential of imidazole derivatives as MCF-7 inhibitors for the treatment of breast cancer. Using Flare’s machine learning, they developed a 3D-QSAR and activity atlas model that classified compound datasets as active or inactive in comparison to a reference drug. Molecular docking analysis discovered active compound interactions with TTK, HER2, GR, NUDT5, MTHFS, and NQO2. The most promising cancer inhibitor was identified as compound C10, paving the way for new approaches to breast cancer treatment.

Hua et al. computationally screened 4,222 anti-cancer compounds against GSK3β. They observed that two potent compounds, BMS-754807 and GSK429286A, had high affinities for binding to GSK3β. These compounds also have promising drug-like properties. This study proposed that BMS-754807 and GSK429286A undergo experimental validation to assess their potential as anti-cancer agents.

Mukhtar et al. screened 9,923 compounds from the ChEMBL database against Tropomyosin-receptor kinase A (TrkA). Among the screened compounds, Delanzomib and Tibalosin, the two leading compounds demonstrated great potential. At 200 ns MD simulations, these compounds demonstrated stable interactions with the TrkA protein. This study proposed that additional research be conducted to determine the viability of Delanzomib and Tibalosin as TrkA inhibitors.

Finally, the authors and editors of this Research Topic hope that the Research Topic of articles will highlight the advances made in the use of computational methodologies for facilitating the design of pharmaceutical compounds directed at various protein targets implicated in cancer management. Furthermore, it is expected that these articles will contribute to a deeper and more comprehensive understanding of the intricate biological processes that underpin cancer, potentially leading to novel therapeutic interventions for this disease. We hope that these articles will serve as a source of motivation, information, and guidance for researchers and scholars working in this field.

